# Mycobiota and Aflatoxin B1 in Feed for Farmed Sea Bass (*Dicentrarchus labrax*)

**DOI:** 10.3390/toxins3030163

**Published:** 2011-02-25

**Authors:** Inês Filipa Martins Almeida, Hermínia Marina Lourdes Martins, Sara Maria Oliveira Santos, Maria Suzana Freitas, José Manuel Gaspar Nunes da Costa, Fernando Manuel d´Almeida Bernardo

**Affiliations:** 1 Direcção Geral de Veterinária, Largo da Academia Nacional de Belas Artes, nº 2, 1249-105 Lisboa, Portugal; Email: ines.almeida@dgv.min-agricultura.pt (I.F.M.A.); susana.freitas@dgv.min-agricultura.pt (M.S.F.); josecosta@dgv.min-agricultura.pt (J.M.G.N.C.); 2 INRB, I.P., Laboratório Nacional de Investigação Veterinária, Serviço de Micologia, Estrada de Benfica 701, 1549-011 Lisboa, Portugal; Email: sarasantos441@hotmail.com; 3 UTL, Faculdade de Medicina Veterinária, CIISA-Pólo Universitário Rua Prof.Cid Santos, Alto da Ajuda, 1300-417 Lisboa, Portugal; Email: fbernardo@fmv.utl.pt

**Keywords:** aquaculture, fungi, fish feed, safety control

## Abstract

Thesafety characteristics of feed used in fish and crustacean aquaculture systems are an essential tool to assure the productivity of those animal exploitations. Safety of feed may be affected by different hazards, including biological and chemical groups. The aim of this preliminary study was to evaluate fungi contamination and the presence of aflatoxins in 87 samples of feed for sea bass, collected in Portugal. Molds were found in 35 samples (40.2%) in levels ranging from 1 to 3.3 log10 CFU∙g^−1^. Six genera of molds were found. *Aspergillus flavus* was the most frequent, found in all positive samples, with a range from 2 to 3.2 log_10_ CFU∙g^−1^. *Aspergillus niger* was found in 34 samples (39.1%), ranging from 1 to 2.7 log_10_ CFU∙g^−1^. *Aspergillus glaucus* was found in 26 samples (29.9%) with levels between 1 and 2.4 log_10_ CFU∙g^−1^. *Penicillium* spp. and *Cladosporium* spp. were both found in 25 samples (28.7%). *Fusarium* spp. was found in 22 samples (25.3%), ranging from 1 to 2.3 log_10_ CFU∙g^−1^. All feed samples were screened for aflatoxins using a HPLC technique, with a detection limit of 1.0 μg∙kg^−1^. All samples were aflatoxin negative.

## 1. Introduction

Nowadays, aquaculture is one of the most important animal husbandry systems, allowing a consistent growth of 1% per year in the last three decades all over the world. This impressive growth has been achieved due to remarkable advances in nutritional, genetic and reproductive management of fish and crustaceans adapted to production in captivity.

Feed safety is an essential factor to assure the productivity of those aquatic husbandries. Safety may be affected by many hazards of biological, physical or chemical origins.

The permanent increase of feed demand for farmed fish enforce the use, each time more frequent, of ingredients of vegetal origins in the formulations, as a consequence of the legal restrictions on the use of meat and bone meal for feeding farmed animals in many geographical areas, especially in the European Union [1].

The increase in the incorporation of vegetal ingredients into fish feed formula enlarges the risk of contaminations coming from that origin, namely fungi and their toxic metabolites, the mycotoxins. 

The natural occurrence of such events has been noticed by many authors. Bautista *et al.* [2] surveyed commercial shrimp feeds in the Phillippines and reported aflatoxin B1 (AFB1) contaminations. In Egypt, Abdelhamid *et al.* [3] detected high levels of aflatoxins (>1000 ppb) in commercial feed used for fish production. The adverse effects in farmed fish attributed to these natural mycotoxin contaminations are not well enough elucidated.

In experimental conditions, sea bass have been exposed to prolonged oral administration of aflatoxins, for 42 consecutive days, and it was verified that this induces a significant increase in serum transaminases and alkaline phosphate activities, and a significant decrease of plasma proteins [4]. The same authors found high levels of AFB1 in fish muscles (≈5 ppb) at the end of the testing period; they concluded that sea bass are vulnerable to AFB1. 

Aflatoxins are secondary metabolites produced in specific ecological conditions by some strains of molds belonging to the groups of *Aspergillus flavus*, *A. parasiticus* and *A. nomius* [5]. Aflatoxins are recognized as inhibitors of nucleic acid synthesis. Depending on the level of exposition, these mycotoxins also decrease protein synthesis, modify lipid metabolism and mitochondrial respiratory pathway; an excessive accumulation of lipids may be noticed in the liver. In trout, prolonged exposure may induce carcinogenic effects: even with doses of 1 ppb of aflatoxin B1 will cause liver cancer. Toxic effects of mycotoxins may differ depending on the age and fish species. Younger fish are more vulnerable; acute aflatoxicoses lesions observed are: pallid gills, impaired blood clotting, anemia, poor growth rates and reduction of growing weight. A prolonged exposure to low concentrations of AFB1 may induce liver tumors, as yellow nodules that also metastases to the kidney; with this scenario, mortality rate is increased [6]. 

Rainbow trout exposed through a diet containing 0.4 ppb of AFB1 for 15 months revealed a greater than 14% probability to develop neoplasia. Feeding trout with a diet containing 20 ppb of AFB1 for eight months resulted in an occurrence of 58% of liver tumors; continuing feeding for 12 months led to an increase of 83% in tumors incidence [7].

Aflatoxins also depress the immune system, making fish more susceptible to bacterial, viral or parasitic opportunist infection. 

In a study conducted during 2002 [8], Nile tilapia was exposed to different concentrations of aflatoxin B1 (0.25, 2.5, 10 and 100 mg∙Kg^−1^ for eight weeks). After two weeks, fish exposed to 2.5 mg∙Kg^−1^ or higher concentrations showed significantly reduced weight gain and hematocrit, compared with the control group. Levels of 10 mg AFB1∙Kg^−1^ in the diet induced excess lipofuscin and irregularly sized hepatocellular nuclei; and diets containing 100 mg AFB1∙Kg^−1^ caused weight loss and severe hepatic necrosis. However, no lesions on spleen, stomach, pyloric intestine, head kidney or heart of fish were observed. Sixty per cent of the exposed fish died within eight weeks.

Sahoo and Mukherjee [9] described reductions of total protein, globulin levels, bacterial agglutination titer, NBT and serum bactericidal activities, enhanced albumin-globulin ratio without change in albumin concentration, in fish exposed to aflatoxin B1, compared to a control group. AFB1 also proved to be immunosuppressive in rohu even at the lowest dose (1.25 mg∙kg^−1^)

Sepahdari *et al.* [10] studied the effects of different levels of AFB1 in growth rate (SGR), weight gain and food conversion ratio (FCR) of *Huso huso*. Fish were fed with diets containing 0, 25, 50, 75 and 100 ppb of AFB1 for three months. SGR was not significantly affected (P < 0.05), however weight gain and FCR were significantly heterogeneous (diets contaminated with 75 and 100 ppb AFB1/kg after 90 days). 

Another relevant aspect related with mycotoxins in fish and crustaceans may come from the fact that hazards or their metabolites may persist in animal tissues and eventually create a risk for human health.

There is no definite correlation between mycotoxin presence in feed and the mycobiota that may be found in this matrix.

Colonization of feed by fungi is relevant when assessing the integrity of the nutrient composition of the feed and the quality of the balanced formula, especially the micro-nutrients. Fungi development enhances loss of nutrients in the feed and compromises its role in farmed fish productivity [11]. Fungi contamination may originate in the raw material feeding stuffs or be a result of a post-manufacturing cross contamination [12].

Mycobiota of feed may be conditioned by technological procedures used in feed production. Some physical treatments, including high pressure and temperature (extrusion) clearly reduce the level of fungi contamination in the finished product [13,14]. Organoleptic losses may also occur [15,16]. 

The most common molds found in feed are *Aspergillus*, *Penicillium* and *Fusarium* genera [17,18,19]. *Aspergilla* are filamentous fungi frequently found in crops, such as cotton seed, peanut meal and corn, wheat, sunflower, soybean and even fish meal. 

The aim of this preliminary study was to search and to characterize the natural mycobiota and aflatoxin contaminations in feed for farmed fish (sea bass) distributed in Portugal. 

## 2. Material and Methods

### 2.1. Sampling Procedure

Eighty-seven aliquots of feed for farmed sea bass, were randomly sampled from two Portuguese feed plants. All samples were aseptically conditioned, sent to the laboratory and tested within 24 hours. Samples were maintained at room temperature.

### 2.2. Mycological Examination

Ten grams of each sample were homogenized for 3 min in 90 mL (10^–1^ suspension) of peptone water (Oxoid, CM 9). Ten-fold dilutions were prepared until 10^–3^.

One milliliter of each diluted suspension was spread onto 4 serial Petri dishes containing two different culture media: Dichloran Rose Bengal Chlortetracycline Agar (DRBCA, Diagnostic Systems-270310) and Sabourad’s agar with choramphenicol (SDA, Diagnostic Systems 221825). Each Petri dish was inoculated with 0.25 mL in the surface of the Agar (1 mL in the 4 dishes); inoculated plates were incubated at 25 °C in the upright position for 5 days [20]. After the incubation period, colonies were counted in the DRBCA plates. Colonies developed in SDA were only considered for eventual identification purposes in case of difficulty to determine fungi genera.

Identification of each fungi colony was determined using macroscopic and microscopic morphology characterizations and taxonomic keys [21,22,23]. 

### 2.3. Aflatoxins Detection and Quantification

Search for aflatoxins was carried out using a standard method [24]. Briefly the technique may be described as follows: a 50 g sample was extracted with a solvent mixture of chloroform stabilized with 0.5% of ethanol (250:25) by shaking for 30 min. After filtration through a folded filter-paper, an aliquot (50 mL) of the filtrate was passed through a Florisil Sep-Pak mini-column (Waters, Milford, MA, USA) previously conditioned with 10 mL of chloroform. The column was rinsed with 10 mL of chloroform, followed by 20 mL of methanol. The toxins AFBs (aflatoxins B1 and B2) and AFGs (aflatoxinas G1 and G2) were eluted with solvent mixture of water and acetone (85 + 15) through C_18_ Sep Pak and the extract was injected in the HPLC. 

The determination of AFBs and AFGs levels in samples extracts was carried out by isocratic reverse-phase liquid chromatography (HPLC) using a LiChrospher 100 RP-18 (5 μm column 25 × 4.6 mm i.d.) EcoPack, with post column derivatization involving bromination, with pyridinum hydrobromide perbromide (PBPB) (Sigma P-3179) and with fluorescence detector. A computing integrator Merck Hitachi (Compaq Deskpro) was used; excitation and emission wavelenghts of λ were 360 nm and 420 nm. The mobile phase was a water-acetonitrile-methanol solution (6 + 2 + 3, v/v/v), and the flow rates were 1.00 mL/min for mobile phase and 0.30 mL/min for reagent PBPB. 

Recovery averages were 90.0%, 90.0%, 87.5% and 85.0% for AFB1, AFB2, AFG1 and AFG2, respectively. The detection limit of the method was 1.0 μg∙kg^−1^. 

### 2.4. Standard Solutions

Standard AFB_1_, AFB2, AFG1 and AFG2 (Sigma–Aldrich) working solutions were prepared using toluene/acetonitrile (98 + 2 by volume) (10 μg/mL). Concentration stock solutions were determined by absorbance at 363 nm. 

## 3. Results and Discussion

Thirty-five samples of feed for farmed sea bass revealed to be contaminated with molds (Table 1). 

**Table 1:** Number and percentage of samples positive for fungi of feed for farmed sea bass.

Matrix	Number of Samples	Number of Positives	Percentage of Positives (%)
Fish feed	87	35	40.2

The total number of fungi varied from 1 to 3.3 log_10_ CFU∙g^−1^ (colony forming units per gram). The only fungi genera detected were *Aspergillus, Penicillium, Cladosporium* and *Fusarium*.

*Aspergillus flavus* was the most frequently found mold in the 35 samples (40.2%), presenting a mean value of 2.7 log_10_ CFU∙g^−1^, ranging between 2.0 and 3.2 log_10_ CFU∙g^−1^ (Table 2). The presence of *A. flavus* in some samples has been pointed to as a potential risk factor to Aflatoxins produced in the feed during storage [6].

**Table 2:** Number and average of mold genera account in feed for farmed sea bass.

Mold	Number of Positive Samples	Percentage of Positives (%)	Average Level of Contamination (log_10_ CFU∙g^−1^) ^(*)^	Range (log_10_ CFU∙g^−1^) ^(*)^
*Aspergillus flavus*	35	40.2	2.7	2–3.1
*Aspergillus niger*	34	39.1	2.2	1–2.6
*Aspergillus glaucus*	26	29.9	1.9	1–2.3
*Penicillium spp.*	25	28.7	2.0	2–2.8
*Cladosporium spp.*	25	28.7	1.9	2–3.3
*Fusarium spp.*	22	25.3	1.8	1–2.3

Note: (*) CFU-colony forming units per g.

*Aspergillus niger* was found in 34 samples (39.1%) at a mean value of 2.2 log_10_ CFU∙g^−1^, ranging from 1 to 2.7 log_10_ CFU∙g^−1^. *Aspergillus glaucus* was found in 26 samples (29.9%), showing colonies levels ranging from 1 to 2.4 log_10_ CFU∙g^−1^.

*Penicillium* spp. and *Cladosporium* spp. were both found in 25 samples (28.7%) in levels that ranged from 2.0 to 2.8 log_10_ CFU∙g^−1^ and 2.0 to 3.3 log_10_ CFU∙g^−1^, respectively. *Fusarium* spp. were found in 22 samples (25.3%), showing levels of contamination that varied from 1 to 2.3 log_10_ CFU∙g^−1^ (Table 2). 

This study identified and enumerated spoilage fungi in less than half of the samples of feed for farmed sea bass, but their toxigenic competence were not determined. 

Jakić-Dimić *et al.* [25] tested 43 samples of feed stuffs used for feed fish production and found high levels of mold contamination (*Aspergillus, Penicillium, Fusarium and Rhisopus*). The highest rank of contamination was registered in corn samples.

Feed stuffs used as ingredients for feed production have been found to be frequently contaminated with fungi in Portugal, as described in many published studies. Results of surveys published in 2007, in crops and feeds for livestock, showed high numbers of positive samples, with levels of contamination that ranged from 1.7 log_10_ to 4.7 log_10_ CFU∙g^−1^ [26,27,28]. These data were considered as inspiration to realize the present study, but results revealed to be very different.

Others results presented by different authors [19] concluded that the levels and frequency of fungi contamination are generically decreasing, taking in consideration the results of longitudinal surveys performed in the last ten years in Portugal. 

Dalcero *et al.* [29] characterized the mycobiota of 130 samples of poultry feed; those samples were contaminated with levels that ranged from 3 to 4.9 log_10_ CFU∙g^−1^ for *Aspergillus spp* and 3.0 to 5.3 log_10_ CFU∙g^−1^ for *Penicilium spp*. These results were in line with those obtained in 2001, screening feed for livestock in Portugal [19]. 

In 2010, a similar study performed by Saleemi *et al.* [30] of 190 samples, showed a lower fungi contamination. 

The other aim of this study was to determine aflatoxins in the analyzed samples. Although the aflatoxins recovery comply with the acceptance limits (98.0, 99.0 and 103.0%), the results show that those mycotoxins were not present in any of the 87 samples. 

Aflatoxins are also common and naturally found in feed stuffs of vegetal origin used as ingredients for feed production. Some authors consider the use of crop of vegetal origin as a rising risk factor for the occurrence of mycotoxicosis in farmed fish and crustaceans [7]. 

Altug and Beklevik (2003) [31] found aflatoxins in many of the 153 samples of fish feed during a three years study (1998 to 2000). In 85 of these positive samples, the levels of contaminations were over 21 µg∙kg^−1^.

Bernardo *et al.* (2009) [32] conducted a study with 27 samples of feed for fish for aflatoxins detection, and found two positive results, with levels of 5 and 6 µg∙kg^−1^.

Jakić-Dimić *et al.* (2005) [25], analyzed 43 samples of raw materials (corn, wheat and barley), used as ingredients for fish feed and found levels of aflatoxin B1 contamination ranging from 5 to 40 µg∙kg^−1^. 

To assess mycobiota and its evolution on the food chain, it is essential to have a tool to manage any intensive or semi-intensive animal production systems. This begins in cultural practices of cereals in the field as well as harvest, transport and storage conditions. Physical, chemical or biological treatments for decontamination are a controversial issue, although some feed additives, namely some authorized adsorbents (Regulation (EC) nº 386/2009) [26], may have some influence on the reduction of mycotoxin absorption at the intestine level; binding to mycotoxins decrease bioavailability and indirectly reduce mycotoxin effects. This question is not adequately solved in regulatory terms. 

## 4. Conclusions

Screening feed for farmed fish for mold contaminations and its toxins is a relevant strategy to assure the safety conditions of these feeds. The presence and the level of potentially toxigenic fungi in fish feed may be relevant to enhance safety guarantees to aquaculture productions. The progressive use of vegetal ingredients in fish feed is an inherent risk for molds and mycotoxin contaminations of an inestimable production factor. 

Results found in the present study may be an expression of an adequate control system to implement in feed plants that produce this kind of feed. However, the specific group of molds that have been found, namely *A. flavus*, *Penicillia* and *Fusaria*, can represent a safety risk if storage conditions, after manufacturing, are compatible with mold growth.
